# Arquivos de Neuro-Psiquiatria, 80 years: part 3 (1983–2002)

**DOI:** 10.1055/s-0046-1827065

**Published:** 2026-08-31

**Authors:** Caio Levi Mendes Costa, Gabriel Ramos de Oliveira Gondim, Emanuela Freire Caetano Davi, Nayana Freire de Almeida Fontes, Bernardo de Gusmão Baumflek Baptista, Thais Graniço Baumflek, Francisco de Assis Aquino Gondim

**Affiliations:** 1 Universidade Federal do Ceará, Faculdade de Medicina, Fortaleza CE, Brazil.; 2 Universidade Estadual do Ceará, Centro de Ciências da Saúde, Fortaleza CE, Brazil.; 3 Hospital Geral de Fortaleza, Fortaleza CE, Brazil.; 4 Centro Universitário Maurício de Nassau, Faculdade de Odontologia, Fortaleza CE, Brazil.; 5 Universidade Federal do Ceará, Faculdade de Medicina, Departamento de Medicina Clínica, Fortaleza CE, Brazil.

**Keywords:** Arquivos de Neuro-Psiquiatria, Brazilian Neurology, History, Medical Journal

## Abstract

**Background:**

Arquivos de Neuro-Psiquiatria (ANP) celebrated 80 years in 2023. We have previously evaluated the publication trends throughout the journal's first 40 years.

**Objective:**

To analyze the publication trends, authorship, and editorial patterns of the volumes 41 to 60 of ANP.

**Methods:**

We analyzed the volumes 41 to 60 of ANP (1983–2002). Data were tabulated independently by five blinded researchers and crossverified by two independent researchers.

**Results:**

From 1983 to 2002, 20 volumes, 91 issues and 2,424 articles were published. We analyzed 2,066 articles after excluding nonresearch papers (1,159 original articles and 770 case reports). Compared with the first 20 years, there was a significant increase in the total number of authors/article (3.96 vs. 2.73, *p* < 0.00001), a significant increase of female authors, from 11 to 28.6% ( *p* < 0.05), and a decrease in the number of pages/article ( *p* < 0.05). Lineu Cesar Werneck, Hélio Afonso Ghizoni Teive, and Milberto Scaff were the most prolific authors. Most of the articles focused on Neurology/Child Neurology subjects, with a progressive decreased percentage of Psychiatry papers and increase in Basic Research contributions. There was a linear increase in the total number of articles from 1983 to 2002 as detected by regression analysis (R ^2^ = 0.9134; *p* < 0.0001) and an exponential increase from 1943 to 2002 (R ^2^ = 0.9046; *p* < 0.0001). Most of the articles were written in Brazilian Portuguese, by authors from Southeastern Brazil (60.2%).

**Conclusion:**

The years 1983 to 2002 marked the transition to the current ANP format: minimal Psychiatry contributions; greater contribution from Southern Brazilian states; and, starting in 1999, greater acknowledgment of sponsorship from research agencies and postgraduate training.

## INTRODUCTION

Arquivos de Neuro *-* Psiquiatria (ANP) a medical journal founded in 1943, has been the leading Brazilian platform in the field of Neurology. 1 We have previously described the characteristics of its first 40 years (1943–1962) as part of a 4-manuscript project aimed to evaluate its history over the first 80 volumes. 2 3

In the first 40 volumes, professor Osvaldo Lange, from the Universidade de São Paulo (USP), served as Chief Editor until his death in 1986, when he was succeeded by professor Antonio Spina-França Netto, in 1987. 2 3 Most of the articles were written in Portuguese and from Southeast Brazil, although included international contributions from Egaz Moniz, Barraquer-Bordas, Bing, Denny-Brown, and Wartenberg. 1 In 1962, the Academia Brasileira de Neurologia (ABN) was founded and in 1970, ANP became the official journal of the Brazilian Academy of Neurology. 3

As we have previously observed, despite the foundation of both Conselho Nacional de Pesquisas, renamed as Conselho Nacional de Desenvolvimento Científico e Tecnológico (CNPq) in 1974, and Coordenação de Aperfeiçoamento de Pessoal de Nível Superior (CAPES) in 1951, as well as Financiadora de Estudos e Projetos (FINEP) in 1967, and Fundação de Amparo à Pesquisa do Estado de São Paulo (FAPESP), founded in 1962, most of the research papers published at ANP were not financed by research grants from those institutions and were clinically-oriented observations (case reports and series). 3 Volumes 21 to 40, coinciding with the Brazilian Military government were also characterized by a transition to modern ANP format and progressive decrease of Psychiatry articles and increase in Basic Science papers. 3 The aim of this study was to continue the investigation of publication trends from 1983 to 2002, taking into account the major historical and scientific developments during this period.

## METHODS

We analyzed all the 20 volumes published by the journal Arquivos de Neuro-Psiquiatria *,* from 1983 to 2002. Each article from the internet-based repository was individually reviewed, as well as all physical editions available at the Professor Jurandir Marães Picanço Health Sciences Library at Universidade Federal do Ceará, as previously described. 2 3 Data from both sources were tabulated by 5 independent and blinded researchers. Subsequently, two researchers cross-verified the collected data.

The following parameters were analyzed in each article: year of publication, number of authors, number of pages, city/state/country of the institution that submitted the article, gender of the authors and article type (original article, case report, practical note, conferences, technical notes). 2 3 Articles from the following categories were excluded from the analysis (except to express general statistical figures), since they frequently did not list the author/institution: book reviews, magazine reviews, *errata* , homages, scientific meetings, and obituaries ( *in memoriam* ). 2 3 We also evaluated the total number of citations from each article as detected by Dimensions AI, available at the ANP Website. 2 3 Descriptive statistical analyses were conducted to detail the most important aspects of the publication (mean ± standard deviation [SD]) and the Chi-squared and two-tailed *t* tests were used to compare the trends in editorial findings throughout the first 60 ANP volumes. 1

## RESULTS

Between 1983 and 2002, 91 issues were published in 20 volumes of the journal. Professor Osvaldo Lange served as Chief Editor until 1986 and was replaced by Professor Spina França in 1987. 1 2 3 During this period, 4 issues were published annually but in 1995, 1997, and 1998, issue 3 was divided in 3A and 3B and from 1999 to 2002, issues 2 and 3 were split into 2A and 2B and 3A and 3B.

A total of 2,424 articles (including all types such as original articles, case reports, book magazine reviews, conferences, *errata* , homages, and obituaries/ *In memoriam* , practical notes, technical notes) were published. Out of that total, 358 were excluded and 2,066 articles were analyzed: 1,159 original scientific articles and 770 clinical case reports. The excluded articles consisted of 88 book reviews, 88 theses, 86 doctoral theses, 34 “ *In Memoriam* ” texts (obituaries or tributes), 16 news and comments, 12 letters to the editor, 9 update articles, 5 correspondences, 5 editorials, 5 homages, 2 abstracts, 2 opinion pieces, 2 seminars, 2 thanks, 1 editor's note, and 1 exchange note.

As such, 8,178 authors were identified in the 2,066 papers, comprising 3.96 ± 2.08 authors/article. There was a statistically significant increase in the number of authors/article in comparison with the first 20 volumes (3.96 vs. 2.73, *p* < 0.0001). The average number of all article subtypes and all original articles published per year during these two decades was 121.2 ± 56.01 and 103.3 ± 45.35, respectively. There was a significant increase in the number of original articles published per year from 1983 to 1992 (65.6 ± 15.5) versus 1993 to 2002 (141 ± 33.77) papers ( *p* = 0.0001).

**Figure 1:**
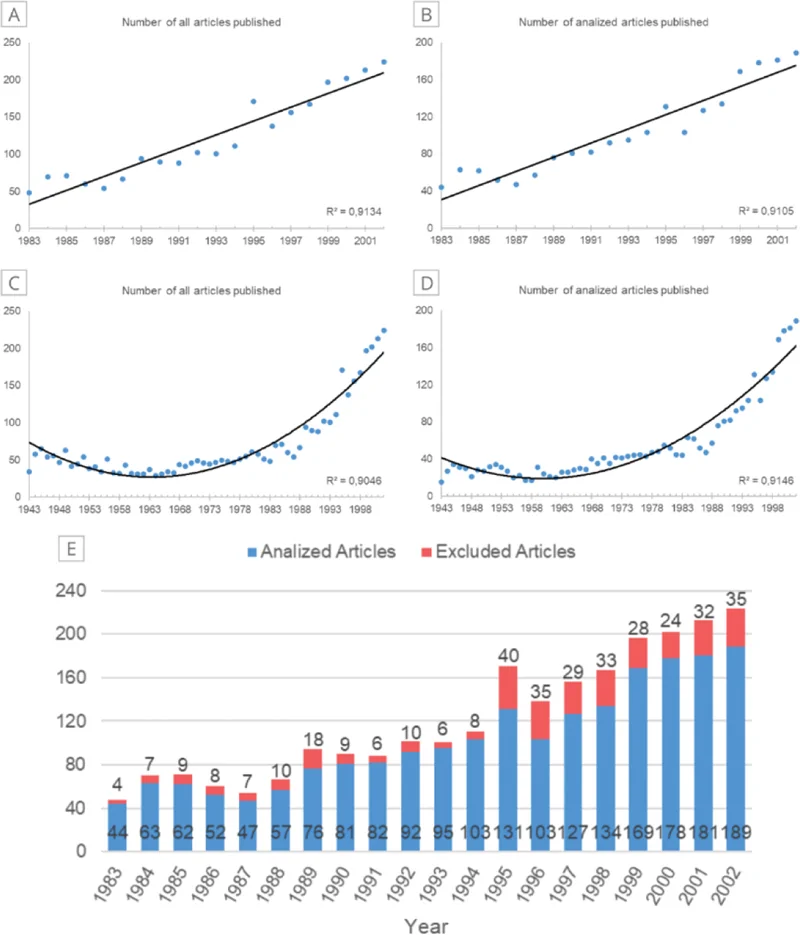
( **A** ) Linear regression analysis of the total number of articles published between 1983 and 2002 (vol. 41–60) and R ^2^ value. ( **B** ) Linear regression of analysis of total articles analyzed from 1983 to 2002 and R ^2^ value. ( **C** ) Polynomial regression analysis of the total number of articles published between 1943 and 2002 (in the first 60 volumes of ANP) and R ^2^ value. ( **D** ) Polynomial regression of total articles analyzed per year from 1943 to 2002 and R ^2^ value. ( **E** ) Number of analyzed (blue) and nonanalyzed (red) articles published per year from 1943 to 2002.

Figure 1 details the trends in the total number of publications and original articles in the first 60 years of ANP publication, including 2 types of regression analysis. Figures 1A and 1B demonstrate a statistically significant growth in both the overall volume of publications (across all subtypes) and analyzed articles' subtypes between 1983 and 2002. This upward trend was confirmed via regression analysis, yielding the equations Y = 9283X-18375 (R ^2^ = 0.9134; *p* < 0.0001) for total publications and Y = 7605X-15049 (R ^2^ = 0.9105; *p* < 0.0001) for original articles; where (X) represents the year and (Y) the number of publications. Figures 1C and 1D further detail the patterns discussed in our prior evaluations, demonstrating an initial trend for decreased number of publications and analyzed subtypes of articles, followed by an exponential increase over the past 20 years, as observed in the quadratic regression of the analyzed articles: Y = 0.1126 x ^2^ − 442.3398x + 434266.945 (R ^2^ = 0.9046; P < 0.0001), for all publications and Y = 0.0803 X ^2^ #x2212; 314.6368X + 308321.9856 (R ^2^ = 0.9146; P < 0.0001) for the analyzed subtypes.

The average number of pages per article of the original articles analyzed during the period of 1983 to 2002 (5.89 ± 2.42) decreased in comparison with 1963 to 1982 (7 ± 4.2; *p* > 0.0001). The average number of pages/article in volumes 41 to 50 was higher in comparison with volumes 51 to 60: 6.38 ± 2.79 versus 5.67 ± 2.19 pages ( *p* < 0.0001). The number of citations per article also greatly increased in 1983 to 2002 (9.85 ± 43.79) in comparison to what we observed from 1943 to 1962 (2.64 ± 5.55), and 1963 to 1982 (2.18 ± 4.2; *p* < 0.001 for both). There was a significant increase in the number of citations per article when comparing the first (41–50) and last (51–60) 10 volumes: 4.51 ± 6.78 versus 12.34 ± 52.62 ( *p* < 0.0001).

Professor Lineu Cesar Werneck stands out as the most prolific author, followed by Professors Hélio Afonso Ghizoni Teive and Milberto Scaff. Table 1 further details the top ten most productive authors in volumes 41 to 60. We also observed a major increase in the percentage of female contributions (author/co-author) in comparison with the first 20 and 40 volumes: 28.6 versus 10.96 and 3.75% ( *p* < 0.0001). Table 1 also details the ten most productive female authors.

**Table 1:** List of the ten most prolific male and female authors from volumes 41 to 60 of ANP (1983–2002)

**Ten most prolific male authors**				
**N°**	**Author**	**Authorships**	**Coauthorship**	**Total**
1	Lineu Cesar Werneck	25	68	93
2	Milberto Scaff	1	46	47
3	Walter Oleschko Arruda	19	24	43
4	Hélio Afonso Ghizoni Teive	20	21	41
5	Luiz Fernando Bleggi Torres	17	21	38
6	Luis Dos Ramos Machado	12	24	36
7	Arthur Cukiert	15	18	33
8	Alberto Alain Gabbai	0	31	31
9	Rubens Nelson Amaral de Assis Reimão	21	10	31
10	Paulo Euripedes Marchiori	8	20	28
**Ten most prolific female authors**				
**N°**	**Author**	**Authorships**	**Coauthorship**	**Total**
1	Maria Valeriana Leme de Moura Ribeiro	10	27	37
2	Rosana Herminia Scola	12	22	34
3	Marilisa Mantovani Guerreiro	9	21	30
4	Leila Maria Cardão Chimelli	4	18	22
5	Newra Tellechea Rotta	3	17	20
6	Lúcia de Noronha	6	8	14
7	Elza Dias Tosta Silva	8	4	12
8	Ana Guardiola	9	2	11
9	Anna Elisa Scotoni	1	10	11
10	Marzia Puccioni-Sohler	7	4	11

Note: *Main authorships + coauthorships.

According to the subject areas, there were 1,164 Neurology (56.34%), 127 Child Neurology (16.07%), 119 Neurosurgery (10.6%), 236 Basic Research (11.42%), and 115 Psychiatry (5.57%) articles. Table 2 further details the subjects of subspecialties of the neurology, neurosurgery, and child neurology articles (each could have more than one subspecialty listed). Table 3 details the 10 articles with the highest number of citations between 1983 to 2002. 4 5 6 7 8 9 10 11 12 13

**Table 2:** List of the main neurological subjects covered in the articles analyzed from 1983–2002

Research Subject	N
Neuroinfection	297
Neurovascular	252
Epilepsy	215
Neuroncology	185
Neuroimmunology	147
Neurogenetics	141
Neuromuscular	138
Movement disorders	135
Others	132
Peripheral neuropathy	113
Behavioral neurology	102
Neurophysiology	90
Neurocognition	84
Myelopathy	78
Cerebrospinal fluid	73
Neuropathology	70
Headache	70
Trauma	44
Sleep disorders	31

**Table 3:** List of the 10 most cited articles in volumes published between 1983 and 2002, with the total number of citations

N°	Author (year)	Title	Citations (n)
1	Bertolucci (1994) 4	The Mini-Mental State Examination in an outpatient population: influence of literacy	1,753
2	Almeida (1999) 5	Reliability of the Brazilian version of the geriatric depression scale (GDS) short form	723
3	Almeida (1998) 6	The Mini-Mental State Examination and the Diagnosis of Dementia in Brazil	324
4	Moll (2001) 7	Frontopolar and anterior temporal cortex activation in a moral judgment task: preliminary functional MRI results in normal subjects	231
5	Moll (2002) 8	The cerebral correlates of set-shifting: an fMRI study of the trail making test	217
6	Bertolucci (2001) 9	Applicability of the CERAD neuropsychological battery to Brazilian elderly	216
7	Brucki (1997) 10	Normative data: category verbal fluency	212
8	Nitrini (1994) 11	Brief and easy-to-administer neuropsychological tests in the diagnosis of dementia	195
9	Assumpção Jr. (2000) 12	Quality of life evaluation scale (AUQEI): validity and reliability of a quality of life scale for children from 4 to 12 years-old	97
10	Fabbri (2001) 13	Validity and reliability of the Portuguese version of the Confusion Assessment Method (CAM) for the detection of delirium in the elderly	93

Abbreviations: AUQEI, Autoquestionnaire Qualité de Vie Enfant Imagé; CAM, Confusion Assessment Method; CERAD, Consortium to Establish a Registry for Alzheimer's Disease; fMRI, functional magnetic resonance imaging; GDS, geriatric depression scale; MRI, magnetic resonance imaging.

Note: Detected by Dimensions AI, available i the ANP Website.

**Figure 2:**
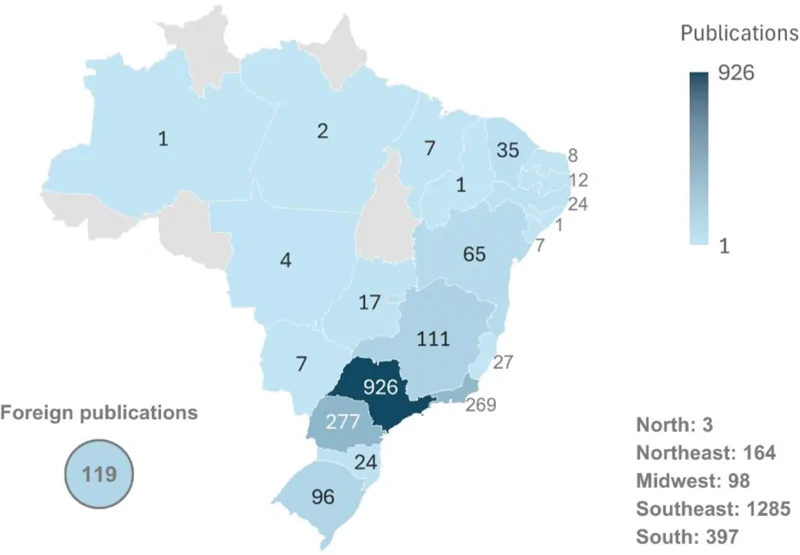
Map of Brazil in 1982 with the number of articles from each Brazilian state and the total number of articles written by foreign authors (international articles).

Figure 2 details the geographical distribution of publications from Brazil. As presented, the origin of the vast majority of articles was Southeastern Brazil (62.2%). Only 3 (0.2%) articles were from Northern (Amazon) Brazilian states, while 4.7% were from the Midwest, 7.9% from the Northeast, and 19.2% from Southern Brazilian states. Furthermore, 119 (5.8%) foreign articles came from the following countries: United States of America (n = 33), Argentina (n = 32), Germany (n = 9), United Kingdom (n = 6), Chile (n = 5); Sweden, Peru, and Spain (n = 4 each); Venezuela, Cuba, and Canada (n = 3 each); Colombia, Italy, and Australia (n = 2 each); Germany, India, Spain, and Portugal (n = 1 each). The vast majority of the articles were published in Portuguese (73.72%). A few articles were published in English (25.31%) and Spanish (0.97%).

## DISCUSSION

As we have previously discussed, the first 40 volumes of ANP (1943–1982) established it as a vital platform to disseminate neuroscientific knowledge within the Brazilian neurological community, quickly surpassing the importance of earlier publications: ‘Arquivos Brasileiros de Psiquiatria,’ ‘Neurologia e Ciências Afins,’ and ‘Revista Neurobiologia’. 1 2 3 The following 20 years (1983–2002) witnessed its continuous role under the guidance of Professor Osvaldo Lange (up to 1986) and Spina-França (1987–2002) after the establishment of a special foundation designed to continue the work of its major guardian (Prof. Osvaldo Lange): Associação Arquivos de Neuro-Psiquiatria Dr. Osvaldo Lange. 14

In the present investigation, comprising our third historical survey, covering the years 1983 to 2002, we disclosed a major (exponential) increase in the number of original papers, keeping the modern article format trends observed in our second analysis, that is to say, shorter articles (compared to 1943–1962) with a larger number of authors. 3 Historically, those new accomplishments coincided with replacement of the Brazilian military government and greater development of clinical postgraduate training (both *lato* and *stricto sensu* ). Most of the papers were published in Portuguese (73.7%), reflecting the needs of the Brazilian neurologists to publish their own accounts in scientific journals circumventing the language barriers from international publications. A quarter of the articles were written in English (25.31%) and only 1% in Spanish (English became the official language of ANP thereafter). The contribution of authors from foreign countries remained stable (5.76%) in comparison with 1963 to 1982 (6.7%) but significantly lower in comparison to the first 20 years of ANP establishment (1943–1962; 17.88%).

Most of the articles were produced in Southeastern and Southern Brazilian states. Compared with the initial 40 years, there was a more important growth in the scientific production from Southern Brazil and a decreased percentage of foreign contributions. 2 3 Those figures are consistent with regional differences in neurological care seen in Brazil. 15 A far greater percentage of female authors (almost 30%) was also observed. The greater contribution of the scientific production within Southern Brazilian states is further reinforced by the presence of male and female authors among the most prolific researchers during the studied period (1983–2002): Lineu Werneck (UFPR, the most prolific author); Hélio Afonso Ghizoni Teive, Walter Oleschko Arruda, and Luiz Fernando Bleggi Torres (also from UFPR); Rosa Herminia Scola (UFPR); Newra Tellechea Rota and Ana Guardiola (UFRS); and Lúcia de Noronha (PUCPR). As can be seen in Table 2 , the first two female authors could also have been included among the top 10 authors (men and women). Among the most prolific female authors, there was also Dr. Elza Dias Tosta Silva, from Brasília (Brazilian Midwest) and past President of the Brazilian Academy of Neurology ( Table 1 ).

As pointed out in two publications from the Chief Editor in 2002, 14 16 the last years of our current analysis were marked by important achievements: increased participation of practically all Brazilian neurological centers, peer review (starting in the 90s), adherence to ICJME guidelines, sponsorship from CNPq, larger paper size (allowing more space for publication), and more issues since 1997. As we previously discussed, despite the foundation of both the CNPq and the CAPES in 1951, FINEP in 1967, and the FAPESP in 1962, 3 only in the 90s did ANP start to publish a section named “Theses” to discuss the MSc and PhDs published in Brazil; and only in 1999 did ANP start to acknowledge sponsorships from Research agencies: 15 in 1999 (CNPq: 9, FAPEMIG: 3, FAPESP: 2, and CAPES: 1), 29 in 2000 (CNPq: 8, FAPESP: 9, CNPq: 8, and other national or international agencies: 9), and 27 in 2001 (CNPq: 8, CAPES: 3, FAPESP: 10, and other national or international agencies: 6). 16

As one can see in Table 3 , different from the first 40 years, the most cited articles dealt with Behavioral Neurology themes, as well as geriatrics, neuroimaging, and neuropsychological, and quality of life. During the first 40 years, papers focused on neuro-infectious diseases were the most highly cited. Overall, ANP papers from 1983 to 2002 were significantly more cited than those from the first 40 years of publication (1943–1982). As can be seen in Table 2 , neuroinfectious diseases continued to be the favorite topic. However, there was a marked growth of papers dealing with cerebrovascular diseases, epilepsy, neuro-oncology, neuroimmunology, and neurogenetics. Following the trends from 1963 to 1982, we did observe a continuous decrease in the proportion of Psychiatry publications (5.6 vs. 17.3 from 1943–1962 and 4.5% from 1963–1982), and a rise in basic science contributions (11.4 vs. 2.36 from 1943–1962 and 4.5% from 1963–1982).

As emphasized in both our previous analyses, 2 3 to avoid the risk of selection bias about specific topics, imprecisions about the first disease descriptions in Brazil, especially considering that not all accounts of case reports are listed in PubMed or have digital object identifiers, we will not elaborate further on publications' individual merit. However, considering the marked rise of knowledge about retroviruses during this period, it is important to highlight the publication of papers about HTLV myelopathy and tropical spastic paraparesis. 17 18

In summary, our analysis of the ANP publication trends from 1983 to 2002 revealed a marked growth in the number and impact of the publications, as well as progressive involvement of most Brazilian neurological centers, especially from Southern and Southeastern Brazil, marking new developmental milestones of the neurological sciences in this country, that is, spreading the development of Neurology from these regions.
